# Low field magneto-tunable photocurrent in CoFe_2_O_4_ nanostructure films for enhanced photoelectrochemical properties

**DOI:** 10.1038/s41598-018-24947-2

**Published:** 2018-04-25

**Authors:** Simrjit Singh, Neeraj Khare

**Affiliations:** Department of Physics, Indian Institute of Technology Delhi, Hauz Khas, New Delhi, 110016 India

## Abstract

Efficient solar to hydrogen conversion using photoelectrochemical (PEC) process requires semiconducting photoelectrodes with advanced functionalities, while exhibiting high optical absorption and charge transport properties. Herein, we demonstrate magneto-tunable photocurrent in CoFe_2_O_4_ nanostructure film under low applied magnetic fields for efficient PEC properties. Photocurrent is enhanced from ~1.55 mA/cm^2^ to ~3.47 mA/cm^2^ upon the application of external magnetic field of 600 Oe leading to ~123% enhancement. This enhancement in the photocurrent is attributed to the reduction of optical bandgap and increase in the depletion width at CoFe_2_O_4_/electrolyte interface resulting in an enhanced generation and separation of the photoexcited charge carriers. The reduction of optical bandgap in the presence of magnetic field is correlated to the shifting of Co^2+^ ions from octahedral to tetrahedral sites which is supported by the Raman spectroscopy results. Electrochemical impedance spectroscopy results confirm a decrease in the charge transfer resistance at the CoFe_2_O_4_/electrolyte interface in the presence of magnetic field. This work evidences a coupling of photoexcitation properties with magnetic properties of a ferromagnetic-semiconductor and the effect can be termed as magnetophototronic effect.

## Introduction

In recent years, the effect of external magnetic field on conventional semiconductors for applications such as spin pumping^1^, Seebeck spin tunneling^2^, spin Hall effect^3^ and spin transfer torque oscillators^4^ has been widely investigated. The current research work in this area focuses to modify the charge transport properties of semiconductors for efficient solar energy harvesting applications. Recently, Sheng *et al*.^5^ demonstrated ~30% enhancement in the photocurrent using a correlated electron oxide La_0.7_Sr_0.3_MnO_3_ system under an external magnetic field of ~6 T. For such system, magnetic field dependence of the correlated gap is suggested for the change in the photocurrents. Pan *et al*.^6^ demonstrated an indirect approach for the magneto-tuning of the photocurrents using magnetic/semiconductor CoFe_2_O_4_/PbZrTiO_3_ composite system. A~13.7% magneto-tuning of the photocurrent under 0.6 T magnetic field is observed which is attributed to the band structure reconstruction due to interfacial stress experienced by PbZrTiO_3_ under applied magnetic field. In the above studied systems a large magnetic field (~Tesla) is required to get significant effect. However, for practical applications appreciable change in the charge transport properties under low applied magnetic field is always desirable. By using conventional semiconductors, it is difficult to achieve this objective as these are not very sensitive to low magnetic field. Compared to conventional semiconductors, ferromagnetic-semiconductors which are more susceptible to magnetic fields can be potential candidates for studying low field magnetic effect on the charge transport properties.

Moreover, in literature most of the work related to magneto-tuning of the photocurrents is reported for photovoltaic applications which make this effect too constrictive. Thus, to widen the applicability of this effect, more attention is required for exploring this effect for other valuable applications. Photoelectrochemical (PEC) splitting of water is an important application of solar energy harvesting for the generation of clean fuel such as hydrogen (H_2_) to overcome the future energy crisis^7–12^. However, to date efficiency of PEC systems is reported to be disappointingly low due to sluggish kinetics of oxygen evolution reaction in the overall water splitting^13,14^. Thus, the fabrication of efficient photoanodes with advanced functionalities is highly desirable to enhance the oxygen evolution reaction in the PEC process^15–20^. In this context, the fabrication of photoanodes using n-type ferromagnetic-semiconductors and tuning of the PEC activity by the application of an external stimulus such as magnetic field can be of great interest.

CoFe_2_O_4_, a ferromagnetic-semiconductor with n-type conductivity, can be a potential candidate for studying magnetic field effect induced tuning of the PEC properties owing to its high magnetostriction^21^, high rate of change of strain with magnetic field^22^, moderate saturation magnetization^23^ and an optical bandgap in the visible light region^24,25^. CoFe_2_O_4_ has inverse spinel structure where, Co^2+^ ions occupy the octahedral sites and half of the Fe^3+^ ions occupy the tetrahedral sites and remaining half of the Fe^3+^ ions occupy the octahedral sites. However, due to a large amount of empty interstitial sites, a small fraction of the Co^2+^ ions can also occupy the tetrahedral sites^22^. The electrical and optical properties of CoFe_2_O_4_ can be tuned depending upon the relative distribution of metal ions (Co^2+^ and Fe^3+^) at the tetrahedral and octahedral sites^26,27^. It can also possess soft magnetic properties at nanoscale due to which low magnetic field will be required to tune the charge transport properties^28–30^. Herein, we report the growth of CoFe_2_O_4_ films using hydrothermally synthesized CoFe_2_O_4_ nanostructures on fluorine doped tin oxide (FTO) substrates and tuning of the PEC properties under low applied DC magnetic fields is demonstrated.

## Results and Discussion

**Figure 1:**
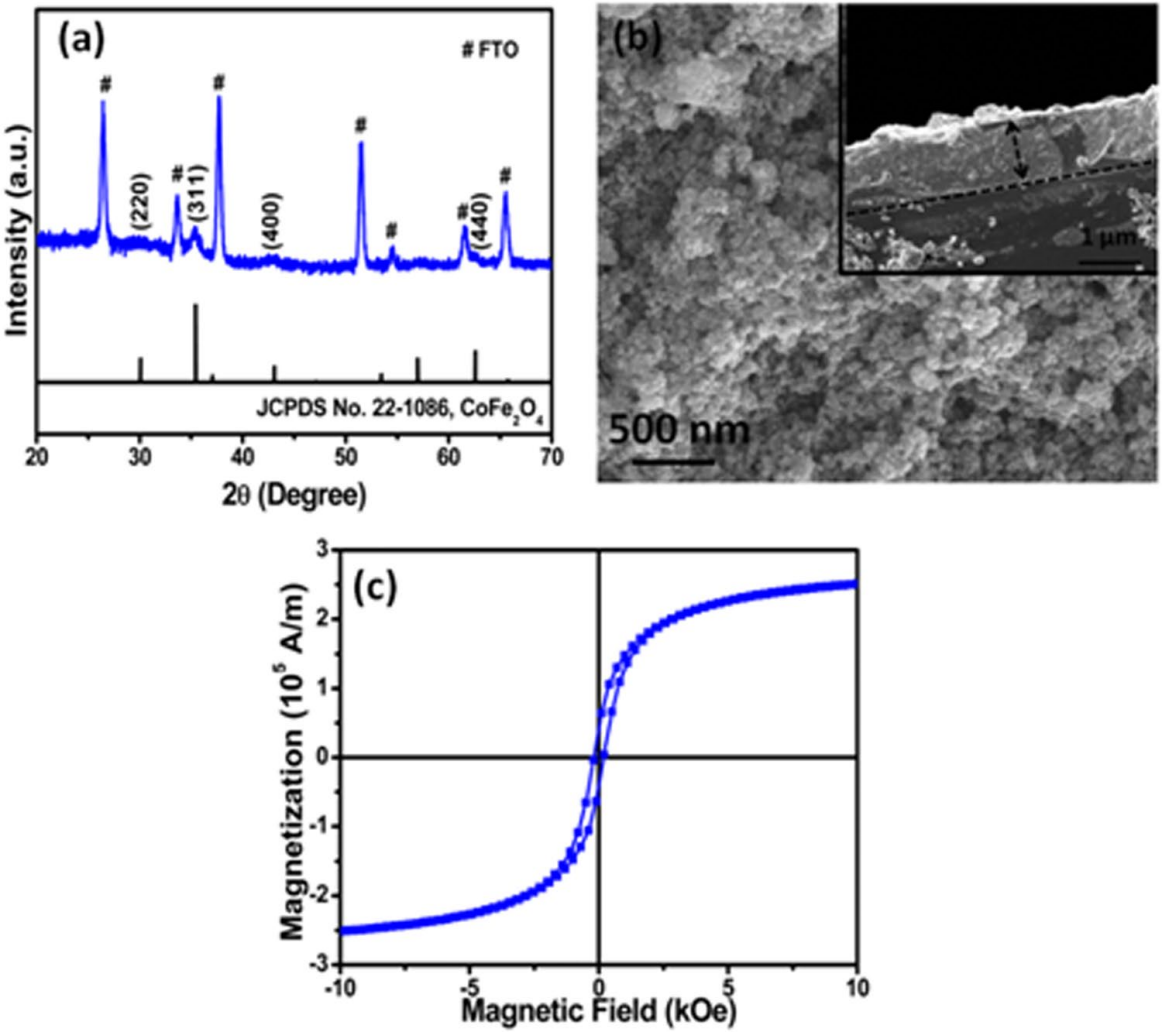
(**a**) X-ray diffraction pattern of CoFe_2_O_4_ nanostructure film, (**b**) top view SEM image of CoFe_2_O_4_ nanostructure film with the inset showing cross-sectional view of the SEM image (**c**) magnetization vs. field characteristic of CoFe_2_O_4_ nanostructure film.

X-ray diffraction pattern (Fig. 1a) and X-ray photoelectron spectra (Fig. S1, Supplementary Information) confirm the single phase formation of CoFe_2_O_4_ nanostructure film. In the XRD data, in addition to the peaks originating from the FTO conducting substrate, all observed diffraction peaks matches well with the standard diffraction data (JCPDS-1086) corresponding to cubic crystal phase of CoFe_2_O_4_ nanostructure film. Figure 1b shows the top view and cross sectional view (inset of Fig. 1b) of scanning electron microscopy image of CoFe_2_O_4_ nanostructure film. Surface morphology of the film shows compact distribution of particles and cross sectional image reveals thickness of the film ~1 µm.

Figure 1c shows the magnetization vs. field (M-H) characteristic of CoFe_2_O_4_ film measured in the bias range of ±10 kOe. M-H loop with coercivity ~180 Oe and saturation magnetization ~2.51 × 10^5^ A/m is observed. A relatively smaller value of coercivity indicates soft magnetic characteristic of CoFe_2_O_4_ film. To confirm type of conductivity in CoFe_2_O_4_ nanostructures, we performed Seebeck measurements (Fig. S2, Supplementary Information) which reveals n-type behavior of the CoFe_2_O_4_ nanostructures.

**Figure 2:**
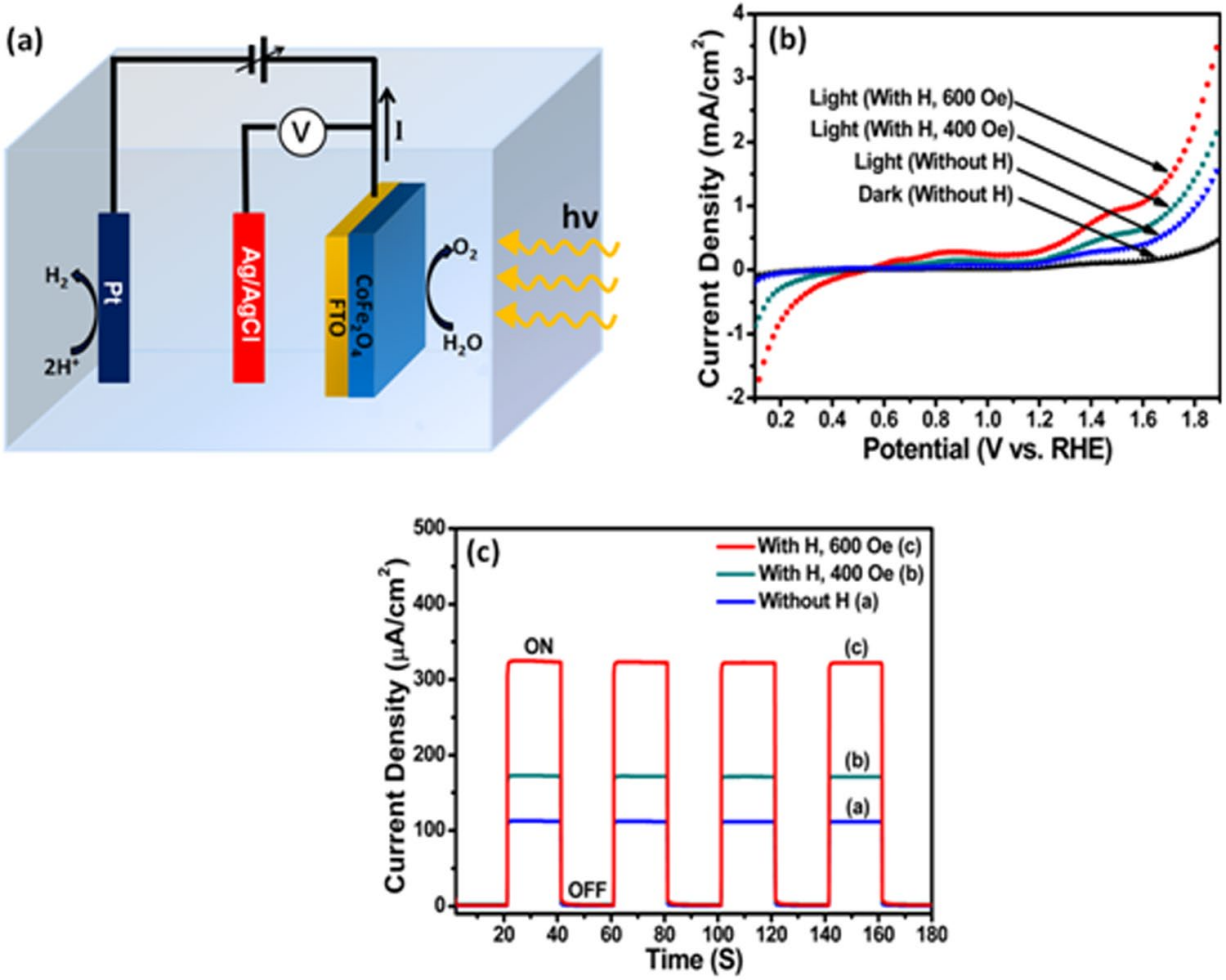
(**a**) Schematic representation of three electrode cell assembly for photoelectrochemical measurements, (**b**) current-potential curves of CoFe_2_O_4_ photoanode measured with and without magnetic field (under 100 mW/cm^2^ UV-vis illumination), (**c**) chronoamperometry results of CoFe_2_O_4_ nanostructure film in the presence of magnetic fields of different strength.

Photoelectrochemical measurements were performed using a three electrode cell assembly with the CoFe_2_O_4_ nanostructure film coated onto FTO substrate as the photoanode, Ag/AgCl and platinum wire as the reference and counter electrodes, respectively and 0.1 M Na_2_S solution as an electrolyte (Fig. 2a). A tungsten halogen lamp with illumination intensity ~100 mW/cm^2^ was used as a light source. In order to study the effect of external magnetic field on the photoanodic behavior of CoFe_2_O_4_ films, magnetic field parallel to the film plane was applied using a permanent magnet assembly. Figure 2b shows the current-potential (J-V) characteristics of as prepared CoFe_2_O_4_ photoanode under dark and light conditions also J-V curves of CoFe_2_O_4_ photoanode under light conditions in the presence of magnetic fields of different strength. In the absence of magnetic field, the J-V curves show a significant photocurrent ~1.55 mA/cm^2^ (at 1.9 V vs. RHE) in CoFe_2_O_4_ film. However, when CoFe_2_O_4_ film was subjected to an external magnetic field of 400 Oe, the photocurrent was found to be enhanced to ~2.14 mA/cm^2^ (at 1.9 V vs. RHE). A maximum enhancement in the photocurrent to ~3.47 mA/cm^2^ (at 1.9 V vs. RHE) was observed with the increase in the magnetic field strength to 600 Oe. With further increase in the magnetic field, no significant improvement in the photocurrent was observed (Fig. S3, Supplementary Information). The observed magnetic field induced change in the photocurrent corresponds to ~123% enhancement in the photocurrent which is significantly higher compared to earlier published works on the magnetic field effect on the photocurrents^5,6^ as well as other effects on the photocurrents (Table S1, Supplementary Information). Figure 2c shows the chronoamperometry results of CoFe_2_O_4_ nanostructure films at a fixed potential of 1.23 V (vs. RHE) in the presence of magnetic fields of different strength. Chronoamperometry results also reveal the same trend in the photocurrent enhancement with magnetic fields. CoFe_2_O_4_ nanostructure photoanode also shows good chemical stability (Fig. S4, Supplementary Information).

The observed enhancement in the photocurrent (J_ph_) can be understood in terms of enhanced generation rate (G) and separation capability of photoinduced charge carriers which in turn depends upon the width of the depletion region (W) at the CoFe_2_O_4_/electrolyte interface and is given by the following relationship^5^;

1 $${J}_{ph} \sim qGW$$

**Figure 3:**
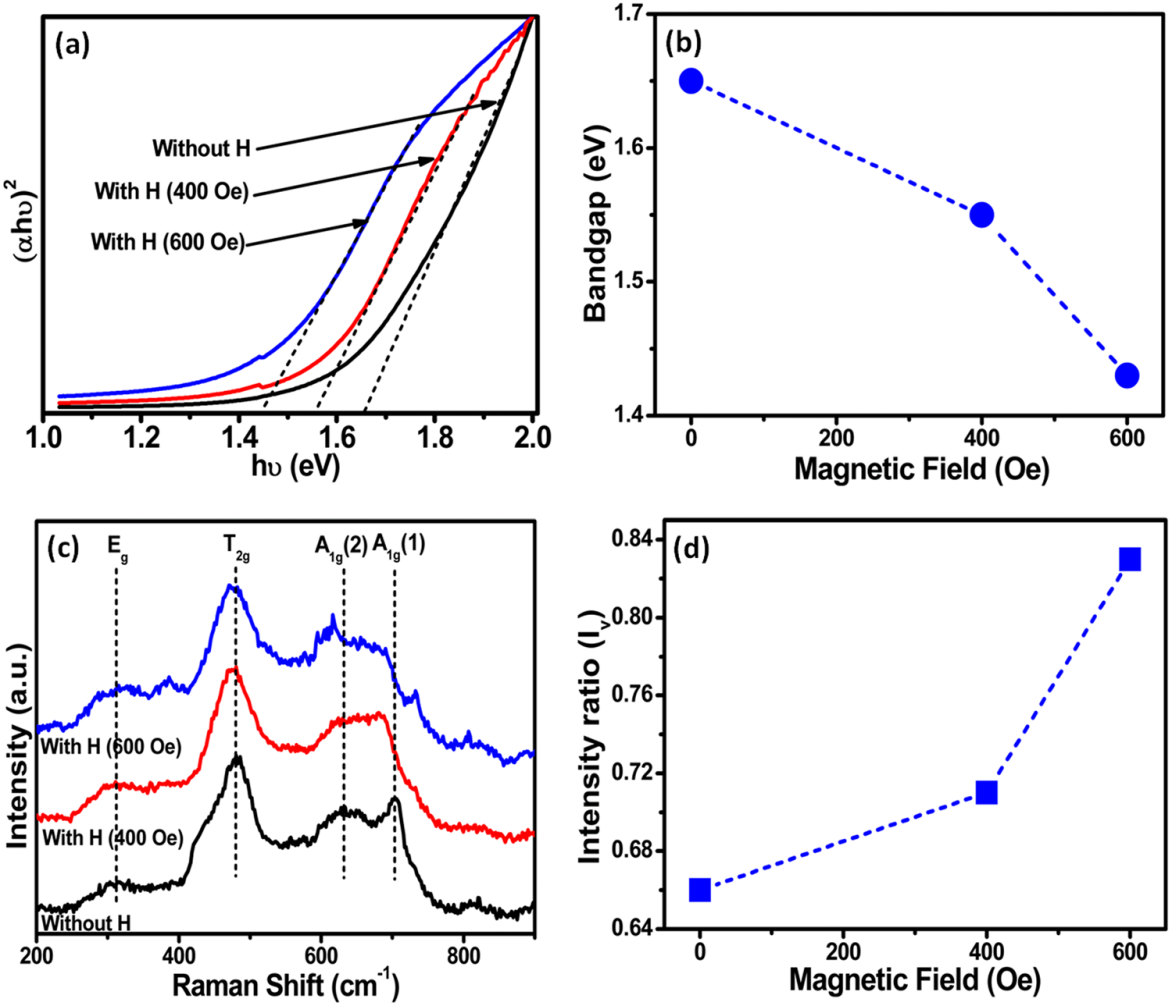
(**a**) Optical bandgap results of CoFe_2_O_4_ nanostructure film under different magnetic field strengths, (**b**) variation of bandgap with magnetic field, (**c**) Raman spectroscopy results of CoFe_2_O_4_ film under different magnetic field strengths, (**d**) variation of intensity ratio (I_v_) with magnetic field.

The enhancement in the generation rate of photoinduced charge carriers is strongly related to the reduction of optical bandgap of CoFe_2_O_4_. Optical bandgap of CoFe_2_O_4_ nanostructure film has been determined using CoFe_2_O_4_ nanostructure film coated onto a quartz substrate. Figure 3a show the optical bandgap of CoFe_2_O_4_ nanostructure film under different magnetic field strengths determined by using the Tauc relation^31–33^;

2 $${(\alpha hv)}^{2}={\rm{A}}(hv-{E}_{g})$$

where, *A* is a constant, *α* is the absorption coefficient, *hv* is the absorbed photon energy and *E*_*g*_ is the optical bandgap.

An optical bandgap of ~1.65 eV is estimated when no magnetic field is applied to CoFe_2_O_4_ nanostructure film. However, the optical band gap reduces to 1.55 eV and 1.43 eV when magnetic field of strengths 400 Oe and 600 Oe are applied, respectively. Figure 3b shows the variation of optical bandgap of CoFe_2_O_4_ nanostructure film with increase in the magnetic field strength.

In CoFe_2_O_4_, crystal field splits d level into ${e}_{g}({d}_{{x}^{2}-{y}^{2}},{d}_{{z}^{2}})$ and ${t}_{2g}({d}_{xy},{d}_{xz},{d}_{yz})$ levels and the optical bandgap is due to d (*e*_*g*_ *level*) to d (*t*_2*g*_ *level*) transitions. The energy width between *e*_*g*_ and *t*_2*g*_ levels is higher at the octahedral sites (Δ_*o*_) as compared to the tetrahedral sites (Δ_*t*_) and is given as^27,34^; ${{\rm{\Delta }}}_{t}=\frac{4}{9}{{\rm{\Delta }}}_{o}$. As, in CoFe_2_O_4_, Co^2+^ ions can reside at the octahedral sites or at the tetrahedral sites and the optical bandgap is strongly dependent upon the relative population of Co^2+^ ions at the octahedral and the tetrahedral sites. It is reported^27^ that the shifting of Co^2+^ ions from the octahedral sites towards the tetrahedral sites results in the decrease in the optical bandgap of CoFe_2_O_4_. Thus, in the present case, it is expected that under the effect of magnetic field the strain gets produced due to magnetostrictive properties of CoFe_2_O_4_. The presence of strain can shift some of the Co^2+^ ions towards the tetrahedral sites from the octahedral sites (probably close to the surface region of CoFe_2_O_4_ nanostructures where super-exchange interactions are supposed to be relatively weak compared to bulk) which in turn will result in the reduction of the optical bandgap of CoFe_2_O_4_. In order to probe the redistribution of Co^2+^ ions at the tetrahedral and the octahedral sites in the presence of magnetic fields, we carried out Raman spectroscopy measurements, which is a powerful technique to probe the cationic distribution in spinel oxides^35^.

Figure 3c shows the Raman spectra of CoFe_2_O_4_ measured with 632 nm excitation wavelength under varying magnetic field strengths. The Raman peaks observed at positions 704, 632, 480 and 310 cm^−1^ correspond to optically active Raman modes (A_1g_ + E_g_ + 3T_2g_) of CoFe_2_O_4_^36,37^. The Raman peak at 632 cm^−1^ corresponds to Co^2+^ ions at the tetrahedral sites and the Raman peak at 480 cm^−1^ corresponds to Co^2+^ ions at the octahedral sites^35,38^. In the presence of magnetic field, a shift in the Raman peaks has been observed as compared to the position of the Raman peaks without magnetic field. A shift in the Raman peaks indicates the presence of strain in the CoFe_2_O_4_ nanostructure film which in corroborated with earlier published reports in literature^39^. A change in the relative intensities of the Raman peaks at 704, 632 and 480 cm^−1^ is also observed which can be due to redistribution of cations at the tetrahedral and octahedral sites. We have calculated the ratio of the intensity of Raman peaks at 632 cm^−1^ and 480 cm^−1^ $({I}_{v}=\,\frac{{I}_{632}}{{I}_{480}})$ which will provide an estimate of the distribution of the Co^2+^ ions at the tetrahedral and octahedral sites. The variation of peak intensity ratio with magnetic field is shown in Fig. 3d. It is evident that the peak intensity ratio increases with increase in the strength of magnetic field which reveals that some of the Co^2+^ ions shift from the octahedral sites to the tetrahedral sites with increase in the magnetic field strength. Thus, shifting of more Co^2+^ ions towards the tetrahedral sites will result in the decrease in the optical bandgap of CoFe_2_O_4_ leading to enhanced generation rate of charge carriers in the presence of magnetic field. Based on the experimental results, a schematic band diagram is proposed (Fig. S5 of the Supplementary Information) which shows the effect of magnetic field on the band positions of CoFe_2_O_4_ resulting in an enhancement in the photocurrent.

**Figure 4:**
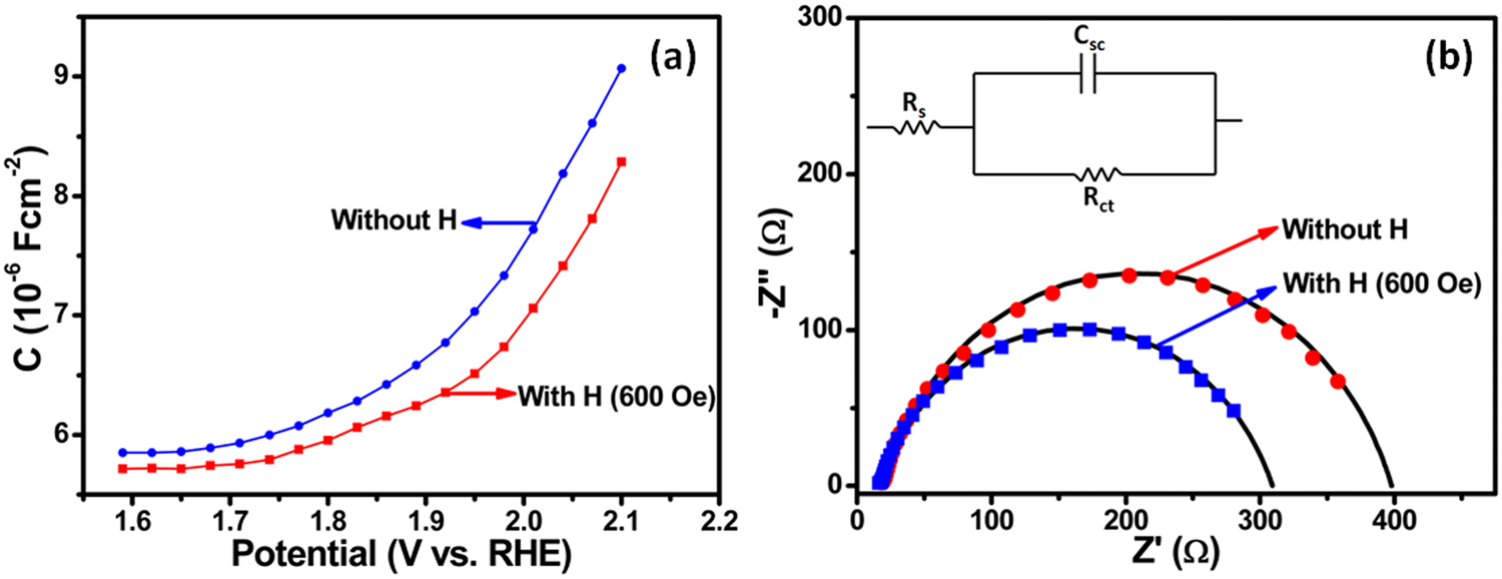
(**a**) Capacitance-voltage curves of CoFe_2_O_4_ nanostructure films measured with and without magnetic field at a fixed frequency of 1 kHz, (**b**) Nyquist plots of CoFe_2_O_4_ photoelectrodes measured under different magnetic field conditions in the frequency range of 100 mHz to 100 kHz (inset of figure shows an equivalent circuit model used for simulations). Solid points (circles, squares) are experimental points and solid lines are simulted curves using an equivalent circuit model.

In order to get further insight into the magneto-tunability of photocurrents, the effect of magnetic field on the junction capacitance (C) was investigated and is shown in Fig. 4a. From the capacitance-voltage (C-V) curves it is clear that C decreases in the presence of magnetic field (600 Oe) which signifies an increase in the depletion region width (W) according to the relation^5^;

3 $$C \sim \frac{\varepsilon }{W}$$

where, *ε* represents the dielectric constant. An increase in the depletion region width results in an effective built-in potential in the depletion region which facilitates the separation of the photogenerated electron-hole pairs and suppresses their recombination rate. To confirm the enhanced separation of the photogenerated charge carriers leading to the enhancement in the photocurrent, we carried out electrochemical impedance spectroscopy (EIS) measurements with and without magnetic field under light irradiation. EIS measures the charge transfer kinetics at the photoelectrode/electrolyte interface. Figure 4b shows EIS Nyquist plots of CoFe_2_O_4_ nanostructure film measured in the presence of magnetic field (600 Oe) and without magnetic field. A semi arc is obtained due to depletion capacitance of semiconductor and Helmholtz capacitance at the electrode surface^40,41^. The semi arc curves are simulated using an equivalent circuit model (shown in the inset of Fig. 4b) with Z-View software and matched with experimental observations^42,43^. The solid lines are the simulted curves. Table 1 shows the estimated values of the parameters R_ct_, R_s_ and C_sc_ from the fitting of EIS Nyquist plots.

**Table 1:** Fitting parameters of the elements used in the equivalent circuit model calculated using Z-View software.

CoFe_2_O_4_ Photoanode	R_s_ (Ω)	R_ct_ (Ω)	C_sc_ (µF)
Without H	17.8	380	8.07
With H (600 Oe)	16	290	7.85

The diameter of the semi arc gives the value of charge transfer resistance (R_ct_) at the electrode/electrolyte interface. It is evident that the charge transfer resistance at the CoFe_2_O_4_/electrolyte interface is smaller in the presence of magnetic field compared to when measured without magnetic field. The decrease in the R_ct_ value confirms the enhanced separation capability of the photogenerated charge carriers resulting in an enhancement in the photocurrent in the presence of magnetic field. We have also performed PEC measurements using Na_2_SO_4_ as an electrolyte and the results are shown in Fig. S6 of the Supplementary Information. The results show that PEC properties are enhanced with the application of an external magnetic field using Na_2_SO_4_ electrolyte also. In literature, charge carrier separation using electric field polarization and piezophototronic effect have been reported^44–46^ however, there is no report on the enhancement in the charge separation efficiency in the presence of magnetic field for PEC applications.

## Conclusions

To conclude, tuning of photoelectrochemical properties of CoFe_2_O_4_ nanostructure film under low external magnetic fields has been demonstrated. It is shown that photocurrent of CoFe_2_O_4_ nanostructure film can be enhanced upto 123% upon the application of magnetic field of 600 Oe strength. The tuning of PEC performance has been correlated to the tuning of the optical bandgap of CoFe_2_O_4_ with magnetic field leading to enhanced generation of the photoexcited charge carriers and also to the enhancement in the depletion width at the CoFe_2_O_4_/electrolyte interface resulting in an enhanced separation of the charge carriers. The tuning of optical bandgap is correlated to the shifting of Co^2+^ ions from the octahedral sites to the tetrahedral sites of CoFe_2_O_4_ in the presence of magnetic field which is confirmed through the Raman spectroscopy measurements. The enhancement in the separation rate of photogenerated charge carriers is confirmed through electrochemical impedance spectroscopy measurements.

## Experimental Section

### Synthesis of CoFe_2_O_4_ nanostructures

**Figure 5:**
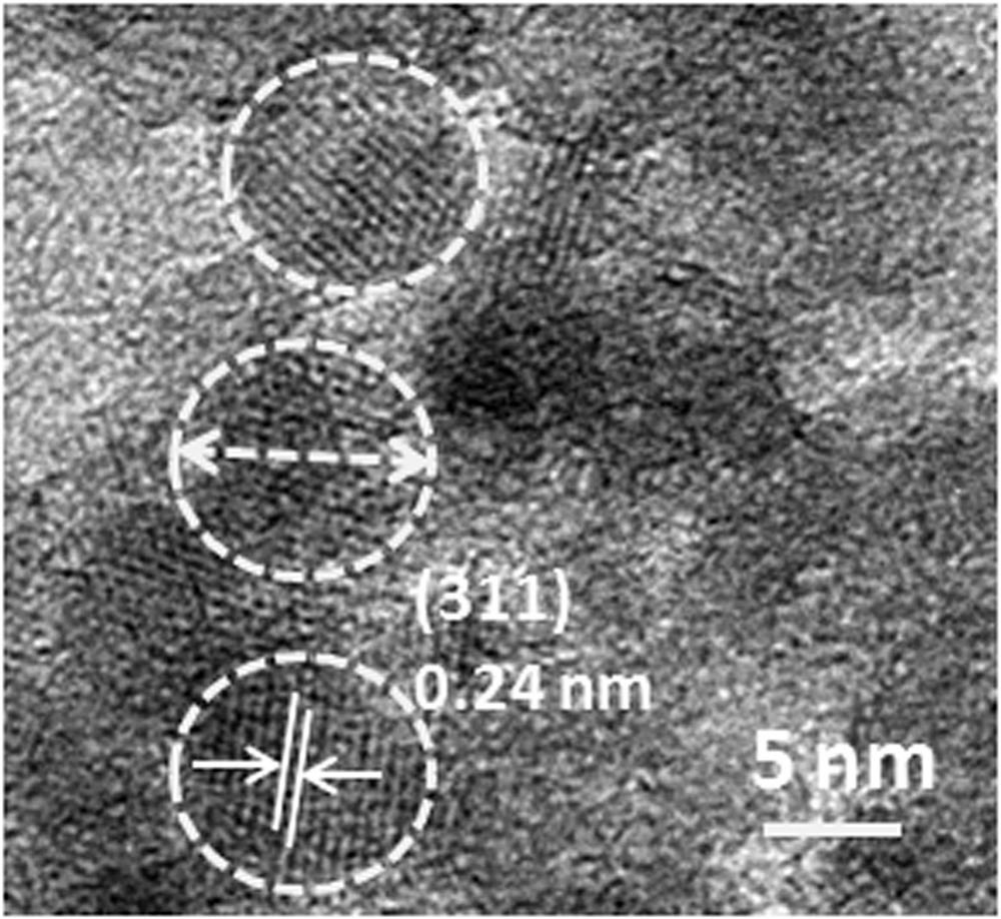
High resolution transmission electron microscopy result of CoFe_2_O_4_ nanoparticles.

For the synthesis of CoFe_2_O_4_ nanostructures, stoichiometric amounts of cobalt nitrate [Co(NO_3_)_2_.6H_2_O, 0.873 g, Merck (99.9%)], iron nitrate [Fe(NO_3_)_3_.9H_2_O, 2.424 g, Merck (99.9%)], sodium hydroxide [NaOH, 2 g, Merck (≥98%)] and ascorbic acid [C_6_H_8_O_6_, 0.141 g, Merck (≥98%)] were mixed in deionized (DI) water. The mixed solution was kept in Teflon lined stainless steel autoclave for heating at 120 °C for 20 hours. After the hydrothermal treatment, resulting CoFe_2_O_4_ nanostructures were washed several times with DI water and dried at 70 °C. Figure 5 shows high resolution transmission electron microscopy (HRTEM) result of as-synthesized CoFe_2_O_4_ nanostructures. The particles of size ~9 nm are formed in the synthesis. The clear lattice fringes indicating good crystallinity with an interplanar spacing of ~0.24 nm is obtained corresponding to (311) plane of CoFe_2_O_4_ nanoparticles^47^.

### Fabrication of photoelectrode

For the fabrication of photoelectrode, the as-prepared CoFe_2_O_4_ nanostructures were dispersed in 20 mL isopropanol and spray coated onto fluorine doped tin oxide coated glass substrate (FTO) heated at ~80 °C. Afterwards, the nanostructure coated FTO substrate was covered with an insulated epoxy leaving an area ~1 × 1 cm^2^ exposed for the photoreaction.

### Characterization

Structural analysis of the CoFe_2_O_4_ nanostructure films was performed using *Rigaku Ultima-IV* X-ray diffractometer with CuKα (1.54 Å) radiation. Magnetic properties of CoFe_2_O_4_ nanostructure films were measured at room temperature using *MicroMag-2900* alternating gradient magnetometer. Optical properties were measured using *Perkin Elmer, Lambda-1050* UV-Vis spectrophotometer. Raman spectroscopy measurements were performed using *Renishaw inVia* Raman spectrophotometer equipped with 632 nm laser source. Surface morphology and thickness of CoFe_2_O_4_ nanostructure film was investigated using *Zeiss EVO 50* scanning electron microscope. Transmission electron microscopy images were performed on CoFe_2_O_4_ nanoparticles using *Technai,G*_*2*_*20 S-Twin* electron microscope operated at 200 kV. X-ray photoelectron spectroscopy measurements were performed using *SPECS* spectrophotometer in ultra high vacuum (~10^−9^ Torr).

### Photoelectrochemical measurements

Photoelectrochemical measurements were performed using a (Zahner Zennium, PP211) potentiostat with a three electrode cell assembly. Nanostructure film of CoFe_2_O_4_ was used as a photoanode, platinum wire as the counter electrode and Ag/AgCl (in sat. KCl, 3.6 M) was used as the reference electrode. A 0.1 M Na_2_S solution was used as an electrolyte solution. A tungsten halogen lamp of intensity ~100 mW/cm^2^ was used as a light source. Current-potential measurements were performed with a slew rate of 10 mV/s. Electrochemical impedance spectroscopy measurements were performed in the frequency range of 100 mHz to100 kHz. Voltage-capacitance measurements were performed at 1 kHz with an AC disturbance of 10 mV.

All experimentally measured potentials vs. Ag/AgCl were converted into reversible hydrogen electrode (RHE) scale using the equation;

$${E}_{RHE}={E}_{Ag/AgCl}+0.059\,pH+{E}_{Ag/AgCl}^{o}$$

where, $${E}_{(Ag/AgCl)}^{o}=0.1976$$ at 25 °C.

## Electronic supplementary material

Supplementary Information
