# Supplementary material for: Low field magneto-tunable photocurrent in CoFe2O4 nanostructure films for enhanced photoelectrochemical properties
Source: Sci Rep. 2018 Apr 25;8:6522. doi: 10.1038/s41598-018-24947-2 (PMC5916887; doi:10.1038/s41598-018-24947-2)
Supplement: Supplementary file 1 — Supplementary Information [file 41598_2018_24947_MOESM1_ESM.doc]

**Supplementary Information**

**Low field magneto-tunable photocurrent in CoFe_2_O_4_ nanostructure films for enhanced photoelectrochemical properties**

Simrjit Singh and Neeraj Khare*

Department of Physics, Indian Institute of Technology Delhi, Hauz Khas, New Delhi-110016, India.

1. **X-ray Photoelectron Spectroscopy Results**

X-ray photoelectron spectroscopy (XPS) measurements have been performed using (SPECS, German company) spectrometer with Mg k_α_ X-ray source (1253.6 eV). Fig. S1(a) shows wide range survey scan of CoFe_2_O_4_ sample.

**
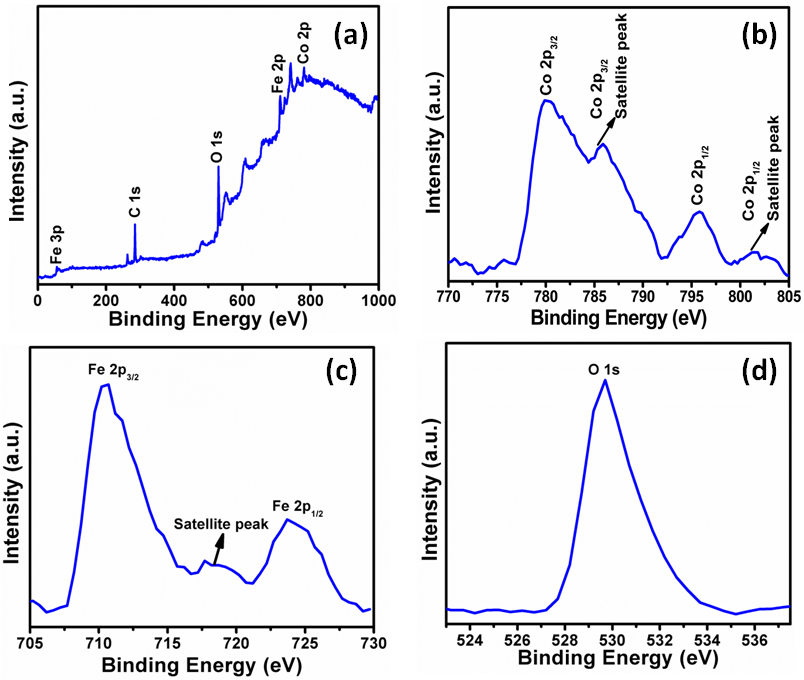
**

Figure S1. X-ray photoelectron spectroscopy scans of CoFe_2_O_4_ sample (a) wide range survey scan and core level XPS scans of (b) Co2p, (c) Fe2p and (d) O1s elements in the CoFe_2_O_4_ sample.

Fig. S1(b) shows core level XPS spectrum of Co2p which exhibits a Co2p_3/2_ and Co2p_1/2_ peaks centered at 779.8 eV and 795.4 eV, respectively along with a characteristic satellite peak located at 787.1 eV which confirm +2 oxidation state of Co atoms in the CoFe_2_O_4_. Fig. S1 (c) shows the Fe2p XPS spectrum which exhibit two peaks located at 710.4 eV and 723.9 eV for Fe2p_3/2_ and Fe2p_1/2_ along with a characteristic satellite peak centered at 718.4 eV corresponding to the +3 valence state of the Fe elements. Fig. S1 (d) shows the O1s core level XPS spectrum. The spectrum exhibit a broad peak located at binding energy 529.7 which can be assigned to the lattice oxygen of CoFe_2_O_4_. The XPS results reveal exact oxidation states of Co and Fe elements which confirm single phase formation of CoFe_2_O_4_ nanostructures^S1,S2^.

1. **Seebeck Measurement Results**

Fig. S2 shows the Seebeck coefficient measurement results of CoFe_2_O_4_ nanostructure. The negative value of the Seebeck coefficient is observed which shows n-type behavior of CoFe_2_O_4_ nanostructures.


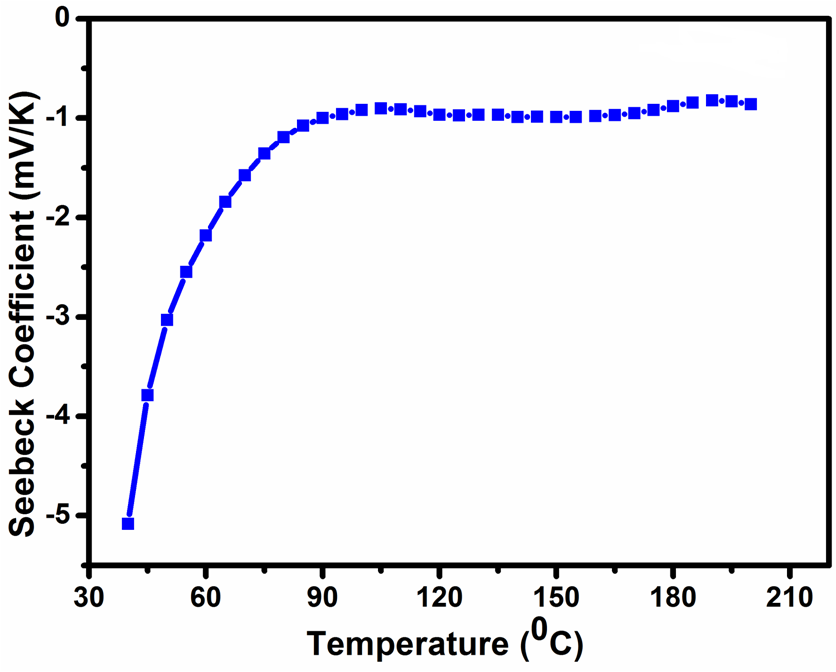


Figure S2. Seebeck measurement result of CoFe_2_O_4_ nanostructures.

1. **Photocurrent and Chronoamperometry Results with Higher Magnetic Field**

Fig. S3 shows current-potential and chronoamperometry results (at 1.23 V vs. RHE) under additional external magnetic field of 700 Oe. The photocurrent obtained under the applied field of 700 Oe is 3.53 mA/cm^2^ whereas, photocurrents obtained under the applied field of 400 Oe and 600 Oe are 2.14 mA/cm^2^ and 3.47 mA/cm^2^ respectively. Thus, there is no significant improvement in the photocurrent with further increase in the magnetic field intensity.


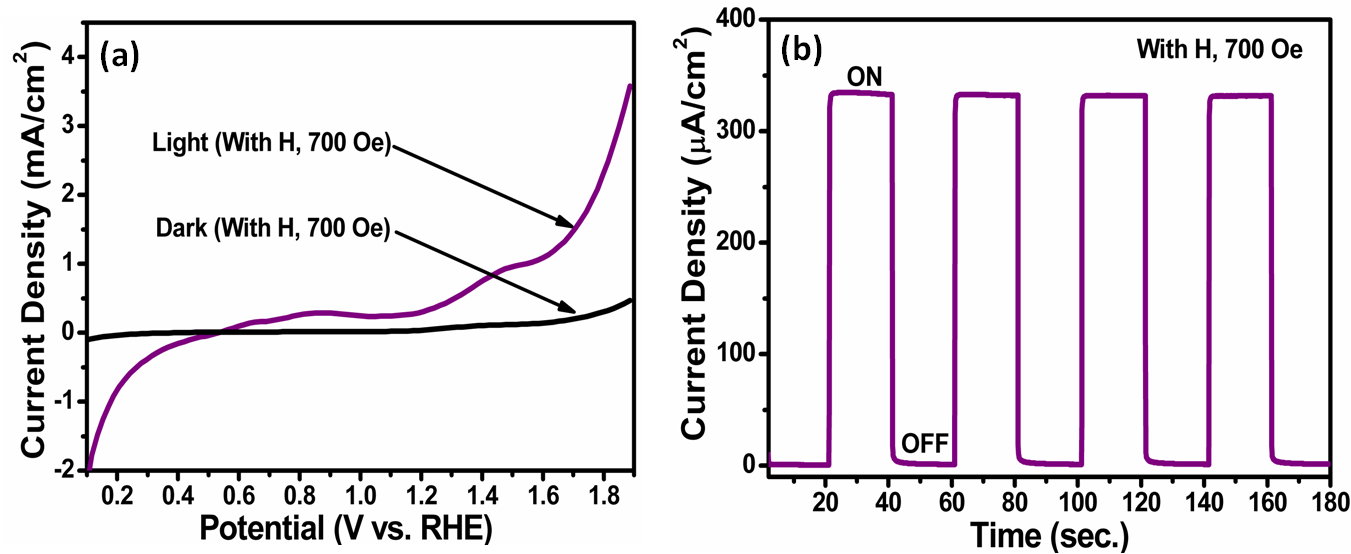


Figure S3. (a) Current-potential curves of CoFe_2_O_4_ photoanode measured with magnetic field strength of 700 Oe, (b) chronoamperometry results of CoFe_2_O_4_ nanostructure film in the presence of magnetic field of 700 Oe.

1. **Stability Test**

Fig. S4 shows chronoamperometry results of CoFe_2_O_4_ nanostructure photoanode measured for 1 hour at 1.9 V vs. RHE to check the chemical stability of the photoanode. A high noise level is observed in the plot but the result shows good chemical stability of the photoanode.

**
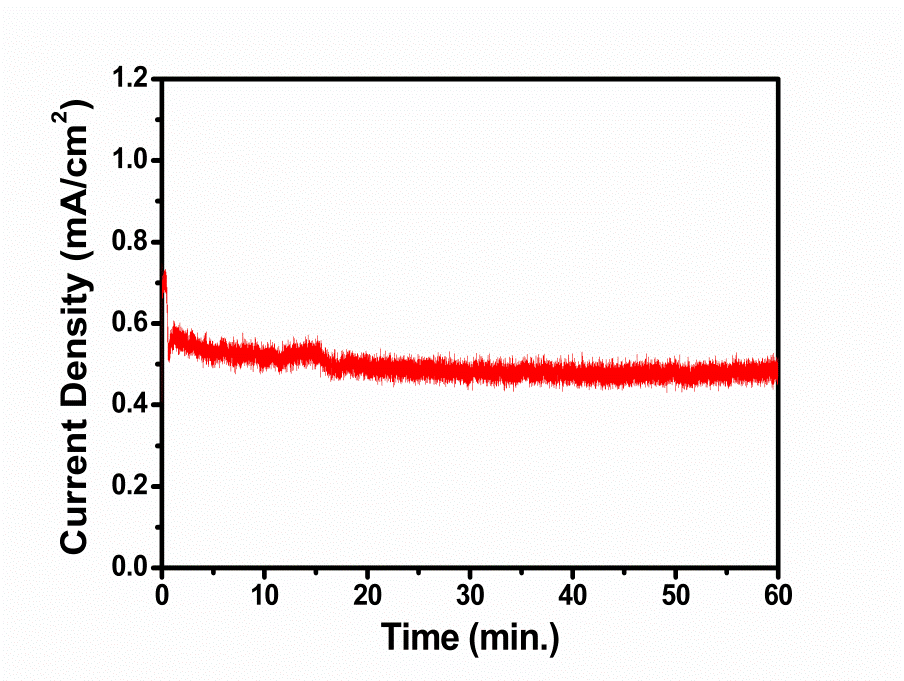
**

Figure S4. Chronoamperometry results of CoFe_2_O_4_ nanostructure photoanode measured at 1.9 V vs. RHE.

1. **Proposed Diagram for the Effect of Magnetic Field on the Band Positions of CoFe_2_O_4_**

Fig. S5 shows a schematic representation of the effect of magnetic field on the band positions of CoFe_2_O_4_. The optical bandgap of CoFe_2_O_4_ nanostructure film reduces with the increase of applied magnetic field. Thus, we assumed that, in the presence of magnetic field the valence band position of CoFe_2_O_4_ is shifted making it closer to the oxidation potential of water. The shifting of valence band towards the oxidation potential of water allows faster transfer of the holes from the valence band to oxidation redox potential thus facilitating the oxidation reaction and resulting in enhanced photocurrent which matches to our experimental observation.


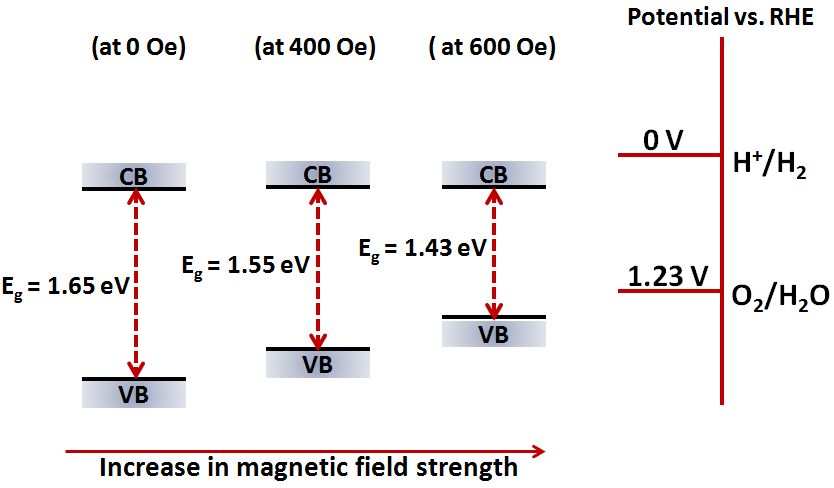


Figure S5. Schematic representation of the band diagram of CoFe_2_O_4_ showing the effect of magnetic field on the band positions.

1. **Photoelectrochemical Results using Na_2_SO_4_ as an Electrolyte**

Fig. S6 show photoelectrochemical results of CoFe_2_O_4_ photoelectrode using 0.1 M Na_2_SO_4_ as an electrolyte. The current-potential curves (Fig. S6a) show enhancement in the photocurrent from 0.128 mA/cm^2^ to 0.371 mA/cm^2^ (at 1.38 V vs. RHE) under the application of 600 Oe magnetic field. Electrochemical impedance spectroscopy results (Fig. S6b) show reduced charge transfer resistance in the presence of magnetic field which confirms enhanced separation of the photogenerated charge carriers leading to enhancement in the photocurrent. Thus, the conclusion of the present work that the photoelectrochemical properties can be enhanced under the application of low magnetic field remains consistent with Na_2_SO_4_ electrolyte also.


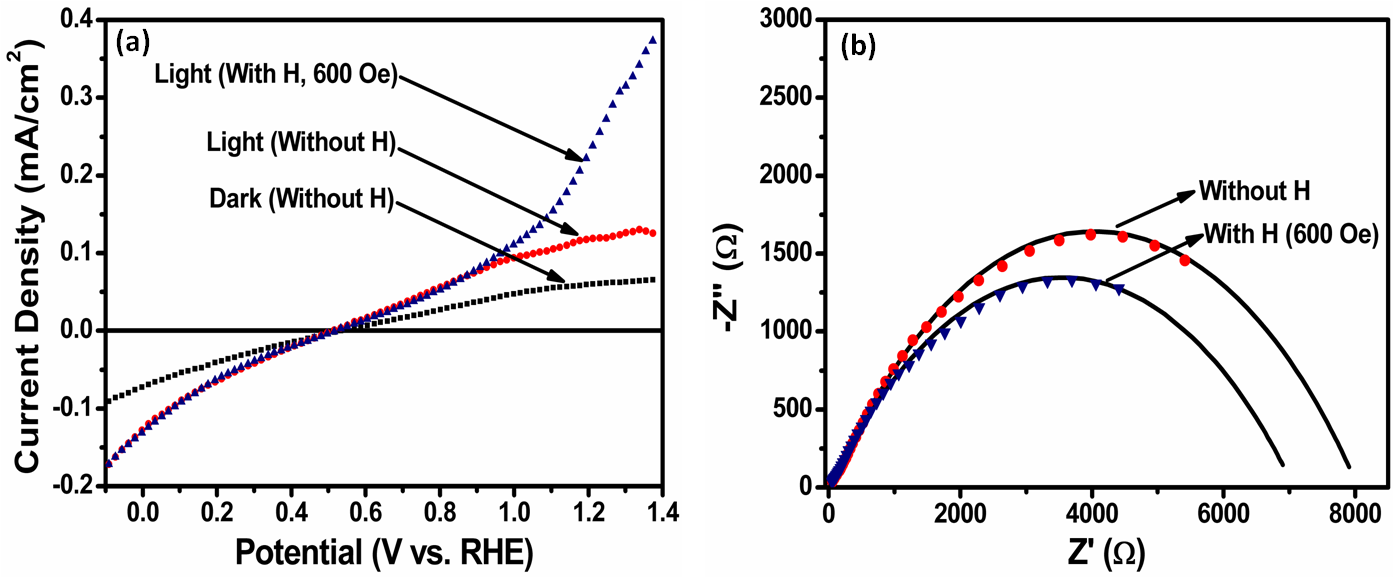


Figure S6. (a) Current-potential curves for CoFe_2_O_4_ photoelectrode measured with and without magnetic field using Na_2_SO_4_ as an electrolyte, (b) Nyquist plots of CoFe_2_O_4_ photoelectrodes measured with and without magnetic field using Na_2_SO_4_ as an electrolyte. Solid points (circles, triangles) are experimental points and solid lines are simulted curves using an equivalent circuit model.

1. **Comparison of the Photocurrent Performance**

A comparison of the enhanced photocurrent performance of our CoFe_2_O_4_ sample to that of other materials reported in the literature has been shown in Table S1. It is evident that the photocurrent enhancement achieved for CoFe_2_O_4_ photoanode under magnetic field is higher than the photocurrent enhancement reported for other photoanode materials.

**Table S1:** Comparison of the enhanced photocurrent performance of CoFe_2_O_4_ photoanode to that of other materials reported in the literature.

| **Sample** | **Initial Photocurrent**  **mA/cm^2^** | **Final Photocurrent**  **mA/cm^2^** | **Photocurrent Enhancement (%)** | **Applied Potential** | **Ref:** |
| --- | --- | --- | --- | --- | --- |
| TiO_2_/BaTiO_3_  (with electrical polarization) | 0.78 | 1.30 | 67 | 1.23 V vs. RHE | [ S3] |
| CaFe_2_O_4_/BiVO_4_ nanocomposite  (relative to pristine BiVO_4_) | 0.58 | 0.96 | 65 | 1.23 V vs. RHE | [S4] |
| Ni(OH)_2_ decorated ZnO  (piezophototronic effect) | 0.482 | 0.740 | 85 | 1.5 V vs. SCE  under 0.2 % strain | [S5] |
| ZnO  (piezophototronic effect) | 0.54 | 0.60 | 10 | 1.5 V vs. SCE  under 0.21 % tensile strain | [S6] |
| NaNbO_3_  (with electrical polarization) | 0.31 | 0.51 | 65 | 1.0 V vs. Ag/AgCl | [S7] |
| Sn doped α-Fe_2_O_3_ (sintered at 800 ^o^C) | 1.24 (nanowire) | 1.86 (nanocoral) | 50 | 1.23 V vs. RHE | [S8] |
| CoFe_2_O_4_  (with magnetic field) | 1.55 | 3.47 | 123 | 1.9 V vs. RHE | Present study |

References:

(S1) Zhou, Z., Zhang, Y., Wang, Z., Wei, W., Tang, W., Shi, J. & Xiong, R. Electronic Structure Studies of the Spinel CoFe_2_O_4_ by X-ray Photoelectron Spectroscopy, *Appl. Surf. Sci.* **254**, 6972 (2008).

(S2) Singh, S., Munjal, S. & Khare, N. Strain/Defect Induced Enhanced Coercivity in Single Domain CoFe_2_O_4_ Nanoparticles, *J. Magn. Magn. Mater.* **386**, 69 (2015).

# (S3) Yang, W., Yu, Y., Starr, M. B., Yin, X., Li, Z., Kvit, A., Wang, S., Zhao, P. & Wang, X. Ferroelectric Polarization Enhanced Photoelectrochemical Water Splitting in TiO_2_-BaTiO_3_ Core-Shell Nanowire Photoanodes, *Nano Lett.* 15, 7574 (2015).

(S4) Kim, E. S., Kang, H. J., Magesh, G., Kim, J. Y., Jang, J. W. & Lee, J. S. Improved Photoelectrochemical Activity of CaFe_2_O_4_/BiVO_4_ Heterojunction Photoanode by Reduced Surface Recombination in Solar Water Oxidation, *ACS Appl. Mater. Interfaces*, **6**, 17762 (2014).

(S5) Li, H., Yu, Y., Starr, M. B., Li, Z. & Wang, X. Piezotronic Enhanced Photoelectrochemical Reactions in Ni(OH)_2_-Decorated ZnO Photoanodes, *J. Phys. Chem. Lett.* **6**, 3410 (2015).

(S6) Shi, J., Starr, M. B., Xiang, H., Hara, Y., Anderson, M. A., Seo, J. H., Ma, Z. & Wang, X. Interface Engineering by Piezoelectric Potential in ZnO-Based Photoelectrochemical Anode, *Nano Lett.* **11**, 5587 (2011).

(S7) Singh, S. & Khare, N. Electrically Tuned Photoelectrochemical Properties of Ferroelectric Nanostructure NaNbO_3_ Films. *Appl. Phys. Lett.* **110**, 152902 (2017).

(S8) Ling, Y., Wang, G., Wheeler, D. A., Zhang, J. Z. & Li, Y. Sn-Doped Hematite Nanostructures for Photoelectrochemical Water Splitting, *Nano Lett.* **11**, 2119 (2011).
